# The Influence of the Dietary Cu-Glycine Complex on the Histomorphology of Cancellous Bone, Articular Cartilage, and Growth Plate as well as Bone Mechanical and Geometric Parameters Is Dose Dependent

**DOI:** 10.1007/s12011-016-0894-x

**Published:** 2016-11-26

**Authors:** Ewa Tomaszewska, Piotr Dobrowolski, Małgorzata Kwiecień, Anna Winiarska-Mieczan, Agnieszka Tomczyk, Siemowit Muszyński

**Affiliations:** 1 Department of Animal Physiology, Faculty of Veterinary Medicine, University of Life Sciences in Lublin, Akademicka 12, 20-950 Lublin, Poland; 2 Department of Comparative Anatomy and Anthropology, Maria Curie-Skłodowska University, Akademicka 19, 20-033 Lublin, Poland; 3 Institute of Animal Nutrition and Bromatology, University of Life Sciences in Lublin, Akademicka 13, 20-950 Lublin, Poland; 4 Department of Physics, Faculty of Production Engineering, University of Life Sciences in Lublin, Akademicka 13, 20-950 Lublin, Poland

**Keywords:** Copper, Cu-Gly, Bone histomorphometry, Mechanical parameter, Adult rat

## Abstract

Copper (Cu) is required for all basic biochemical and physiological processes. The objective of this study was to compare the effect of two different chemical forms (sulfates and glycinate chelates also below the recommended dose) of Cu administered to adult rats on the biomechanical and morphometric properties of femur. Male rats at the age of 12 weeks were used in the 12-week experiment. The control diet provided the required Cu level from sulfate (S-Cu), and the other diets were supplemented with Cu-glycine complex. The Cu-Gly-treatment, irrespective of its concentration, did not influence the bone mass and length. The Cu-Gly-treatment in 100 and 75% of daily demand increased mechanical endurance. The Cu-Gly-treatment (regardless of its concentration) increased the real bone volume in epiphysis and decreased the total thickness and zone I of the articular cartilage compared to the control group supplemented with S-Cu. The Cu-Gly-treatment enhanced the content of proteoglycans (except the OG50 group). Dietary Cu given to adult rats in the Cu-Gly complex covering the daily demand in 75% exerted a positive effect on bone metabolism and appeared to be the most effective among the investigated doses of the organic form.

## Introduction

Copper (Cu) is the third most abundant essential trace element in the body of animals and humans, besides iron and zinc. The liver and other organs contain the highest amounts of Cu among all the tissues, in contrast to the lowest content in bones. The amount of Cu in the body alters during the lifetime in living organisms [1, 2]. A comparative study in long-living mammals demonstrates that Cu concentrations in cartilage with adjacent compact bone is the highest in foxes and the lowest in humans, while in spongy bone they are the highest in dogs and the lowest in foxes [3]. It is important that Cu plays important functional roles in bone metabolism and turnover. It is known to be essential for normal growth and development of the skeleton in humans and in animals [4]. As an essential cofactor, Cu is needed for the action of various enzymes, including lysyl oxidase, i.e., a Cu-dependent enzyme that acts only on extracellular collagen molecules regulating their total enzymatic cross-link formation in connective tissue. The enzyme mediates the final step in the biosynthesis of, first of all, collagen as well as elastin and keratin (hair keratinization) and normalizes the deposition of calcium and phosphorus in the bones [5]. Studies on Cu supplementation in humans and animals indicate that a deficiency of Cu leads to bone loss and reduced bone mass, resulting in a decrease in its mechanical strength and subsequent fractures [6]. The possible underlying causes of these changes are functional defects of osteoblasts (bone tissue-forming cells), while the activity of osteoclasts (bone tissue removing cells) remains unchanged [4]. The process of bone formation requires an adequate and constant supply of nutrients, including Cu [7]. Since bones undergo continuous remodeling, an inadequate supply of Cu could not support the formation phase of bone remodeling and this could lead to the clinical risk of osteoporosis in later life, resulting in problems with locomotor function. Moreover, in humans, copper supplementation for 2 years was associated with reduction in bone loss in perimenopausal and postmenopausal women [7]. Thus, the role of Cu in the metabolism of connective tissue is so prominent that one can speculate that inadequate Cu dietary intake could be an important factor in the etiology of bone loss or osteoporosis and even osteoarthritis in adults.

Copper is one of the microelements used in combination with amino acids. Feed grade chelates based on glycine seem to be a good solution to supplement microelement deficiency in feed rations. Glycine, as a chelate component, is the most easily assimilable amino acid, which, with its wide range of applications, additionally improves the values of this additive [8].

The objective of this study was to compare the effect of two different chemical forms (sulfates and glycinate chelates) of Cu administered in feed mixtures to adult rats on the biomechanical and morphometric properties of femur. The next aim was to evaluate the effects of the diet containing two levels of Cu in the Cu-glycine complex, below the recommended dose (75 and 50% of daily demand), on bone metabolism and cartilage morphology in adult rats. The present study was also performed to test the hypothesis that Cu long-term treatment in a lowered amount in relation to the daily demand could not negatively influence the metabolism of the skeletal system of adults. For this purpose, the present study involved a combined use of multiple methods, i.e., mechanical endurance and light microscopy in combination with a histochemical method to characterize the role of Cu in adult nutrition.

## Materials and Methods

The experimental procedures used throughout this study were approved by the Local Ethics Committee on Animal Experimentation of University of Life Sciences of Lublin, Poland. The rats were maintained in an animal house according to the guidelines of this committee. All efforts were made to minimize the number of animals used as well as their suffering.

### Animals, Breeding, and Experimental Design

Male adult Wistar rats (*n* = 48) at the age of 12 weeks at the start of the experiment were used in the experiment lasting 12 weeks (excluding the acclimatization in the first week). Clinically healthy rats were individually kept in Macrolon cages at 21 ± 1 °C and 55% humidity and 12-h light and dark cycles. The rats were randomly divided into the control and three experimental groups (each *n* = 12), depending on the different levels of organic Cu supplementation. All the animals had free access to distilled water (no Cu) and were fed ad libitum*.* The composition of basal diet comprised crude protein min. 14.5%, crude fat min. 1.5%, crude fiber min. 5%, and ash 10%. The content of vitamin and mineral premixes of the diet is presented in Table 1. The control group was fed a standard diet (LSM, Agropol S.J., Motycz, Poland), which provided the required Cu level for rats in an inorganic form (the IN group; 5 mg/kg of body weight per day from sulfate (S-Cu; CuSO_4_; Avantor Performance Materials Poland S.A., Lublin, Poland)) [9]. In turn, the other animals (the OG100 group) were fed the same standard diet, which provided the required Cu level for rats in an organic form as a Cu amino acid chelate (5 mg/kg of body weight per day from the Cu-glycine complex; Cu-Gly; ARKOP Sp. z o.o., Bukowno, Poland). The rats from the OG75 group received standard diet, which provided a lowered Cu level in an organic form covering 75% of daily demand (3.75 mg/kg of body weight per day). The rats from the OG50 group received standard diet, which provided a lowered Cu level in an organic form covering 50% of daily demand (2.5 mg/kg of body weight per day). Water and feed consumption was measured weekly. At the end of the experiment, the rats were fasted for 24 h and euthanized one by one with carbon dioxide inhalation and by dislocation of the spine.

**Table 1:** The composition of vitamin and mineral premixes of the diet (per kilogram dry matter) fed to rats during the study

Components	Per 1 kg of premix
Manganese (mg)	5000
Iron (mg)	5000
Zinc (mg)	2500
Iodine (mg)	75
Pantothenic acid (D-calcium pantothenate) (mg)	900
Retinol acetate (UI)	800,000
Cholecalciferol (UI)	100,000
Tocopherol (mg)	4964
Menadione sodium bisulphite (mg)	300
Riboflavin (mg)	600
Pyridoxine HCL (mg)	60
Cyanocobalamin (mg)	1.2

### Plasma Biochemical Analyses

Blood samples were collected (two times, after 6 weeks of the duration of the study and at the end) carefully for blood plasma biochemical analysis using standard venipuncture of the heart. The plasma was immediately separated by centrifugation and stored at −25 °C for further analysis. The plasma concentration of Cu, Fe, and Zn was determined by the colorimetric method using a Metrolab 2300 GL unit (Metrolab SA, Buenos Aires, Argentina) and ready-made sets produced by the company BioMaxima (Lublin, Poland).

### Bone Collection and Analysis

The bone length and weight were measured after removal of soft tissues from the left femora. Each bone was wrapped in gauze soaked in isotonic saline and stored at −25 °C for further analysis.

Geometric properties such as the cross-section area (A), mean relative wall thickness (MRWT), and cortical index (CI) were estimated on the basis of horizontal and vertical diameter measurements of the mid-diaphyseal cross section of bone using a method described previously [10, 11].

The mechanical properties of the femur were determined for the bones after 3-h thawing at room temperature using the three-point bending test. The mechanical properties were examined on a Zwick Z010 universal testing machine (Zwick GmbH & Company KG, Ulm, Germany) equipped with a measuring head (Zwick GmbH & Company KG, Ulm, Germany) with an operation range up to 10 kN, linked to a computer with TestXpert II 3.1 software (Zwick GmbH & Company KG, Ulm, Germany) registering the relationship between force perpendicular to the longitudinal axis of the bone and the resulting displacement. The distance between the supports was set at 40% of the total bone length. The measuring head loaded bone samples at a constant speed of 10 mm/min [12]. The ultimate strength was determined as a point at which disintegration of tissue occurred [10].

After removal of soft tissues, the joint with no visible lesions and degenerative changes was opened and full-thickness cartilage was excised with bone specimens. Cylindrical 20-mm thick samples (cartilage and bone) were taken from the same anatomical position in the rats’ knee joint, i.e., from the middle of the lateral femoral condyle (containing epiphysis and metaphysis) immediately after euthanasia. Sagittal sections of the cartilage and bone were cut perpendicular to the articular surface [13]. The tissue samples were subjected to typical common histology and microscopy procedures [14]. Four-micrometer thick sections were cut. Goldner’s trichrome staining was used to assess the morphology of the growth plate cartilage and the articular cartilage [14, 15]. Additionally, articular cartilage proteoglycans were stained with Safranine O (SO) [11]. Microscopic bright-field images were collected using a confocal microscope Axiovert 200M (Carl Zeiss, Jena, Germany) equipped with a camera AxioCam HRc (Carl Zeiss, Jena, Germany) and a halogen lamp [11].

The analysis of the collected images was performed with the use of graphical analysis software Olympus cellSens version 1.5 (Olympus, Tokyo, Japan). The thickness of the following zones reserve (I; cells exist singly or in pairs separated by an abundant extracellular matrix), proliferation (II; chondrocytes assume a flattened shape and are arranged in longitudinal columns; they enlarge and divide), hypertrophy (III; the cell size abruptly increases and the columnar arrangement is less regular), and ossification (IV; the region where the transition from cartilage to bone occurs with degeneration and death of chondrocytes) was measured at four sites along the growth plate cartilage and an average was calculated [13, 16]. Similarly, the thickness of the main zones of the articular cartilage, i.e., horizontal (superficial surface I; chondrocytes are small and flattened parallel to the surface), transitional (II; chondrocytes are large and round, occur singly or in isogenous groups), radial (III; spherical chondrocytes lie in columns), and calcified zone (IV; a zone that rests directly on the subchondral bone) was measured [17].

The bone volume (BV) and tissue volume (TV) were measured in the photographs of the bone tissue sections using the pixel count, and the relative bone volume (BV/TV%) was assessed. Other parameters examined for trabecular bone (epiphysis and metaphysis) included trabecular thickness (Tb.Th) and trabecular separation (Tb.Sp) defined as the distance between the edges of adjacent trabeculae (measured directly) [12, 14].

After evaluating the strength and structural properties, the bones were defatted, dried to constant mass, and finally mineralized in a muffle furnace at 600 °C [18]. The content of Cu in the bones was determined by atomic absorption spectrometry using a Unicam 939/959 apparatus. The content of Cu in the bone was calculated as the content of these components in crude ash.

### Statistical Analysis

All the results are expressed as means ± SD (standard deviation). Differences between the means were tested with the one-way ANOVA and post hoc Tukey’s test as the correction for multiple comparisons. Normal distribution of data was examined using the W. Shapiro-Wilk test and equality of variance was tested by the Brown-Forsythe test. A *P* value of less than 0.05 was considered statistically significant. All statistical analyses were carried out by means of Statistica 12 software (StatSoft, Inc., Tulsa, OK, USA; http://www.statsoft.com).

## Results

### Body Mass, Feed and Water Consumption

The initial values of body mass of the control rats and animals treated with the organic Cu form (regardless of the amount of daily demand) were similar. After 6 weeks of the treatment, the rats supplemented with the organic form of Cu, irrespective of its amount, weighed more than the animals from the control group administered with the inorganic form. At the end of study, after 12 weeks of the treatment, only rats from the OG75 group were heavier, compared to the other groups (Table 2). Weekly water and feed consumption did not differ among all the groups, irrespective of the form of the supplemented Cu and its concentration.

**Table 2:** Mean weekly water and daily feed consumption, the body weight (initial, after 6 weeks, and 12 weeks as final body weight) in control rats and treated with different levels of Cu-Gly

Group	*n*	Body weight, g			Water consumption (ml)	Feed consumption (g)
		Initial body weight (g)	After 6 weeks	After 12 weeks		
CONT	12	356.8 ± 28.4	396.2^b^ ± 26.9	464.2^a^ ± 39.8	85.3 ± 14.8	163.9 ± 13.8
OG100	12	352.4 ± 24.9	453.3^a^ ± 21.1	476.9^a,b^ ± 30.6	83.5 ± 12.6	165.6 ± 17.3
OG75	12	357.2 ± 12.9	467.4^a^ ± 19.9	499.8^b^ ± 6.0	84.7 ± 16.2	167.1 ± 17.3
OG50	12	356.9 ± 10.8	453.2^a^ ± 22.0	488.6^a,b^ ± 15.3	85.8 ± 12.3	166.2 ± 13.5
SEM		2.883	9.601	10.033	2.120	1.884
*P* value		0.930	0.001	0.031	0.855	0.996

Data given are mean ± SD. CONT—the control group received Cu in 100% of daily demand from sulfate (S-Cu), OG100—the group received Cu in 100% of daily demand from Cu-Gly, OG75—the group received Cu in 75% of daily demand from Cu-Gly, OG50—the group received Cu in 50% of daily demand from Cu-Gly

^a,b,c^Mean values in rows with different letters differ significantly at *P* < 0.05

### Cu, Fe, and Zn Content in Blood Plasma

**Fig. 1:**
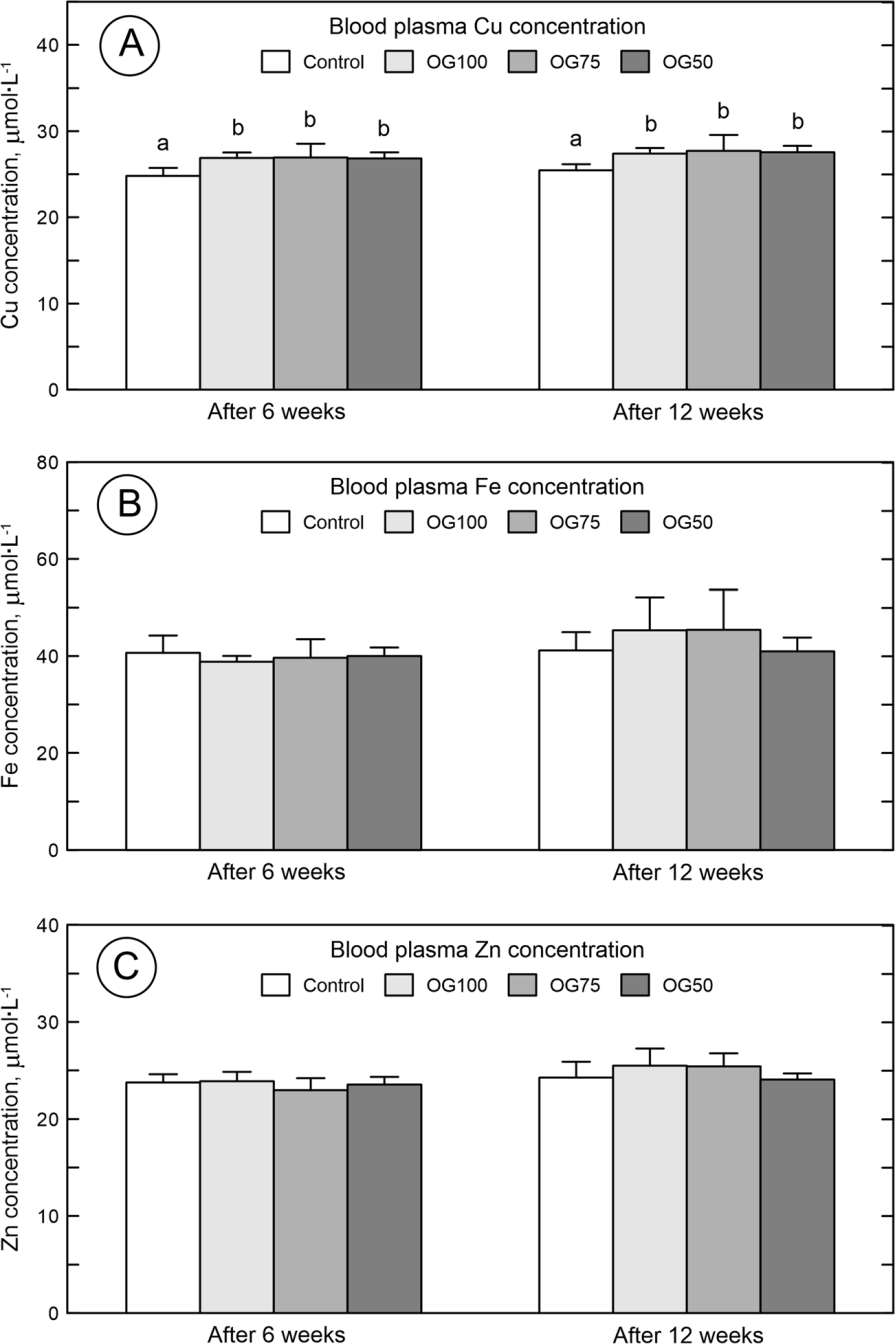
The plasma concentration of copper (Cu) (**a**), iron (Fe) (**b**), and zinc (Zn) (**c**) in blood plasma of 24-week-old rats treated with Cu in organic (Cu-Gly) and inorganic form (S-Cu) after 6 and 12 weeks of the duration of the study. Data given are mean ± SD, *P < 0.05. CONT—the control group received Cu in 100% of daily demand from sulfate. OG100—the group received Cu in 100% of daily demand from Cu-Gly. OG75—the group received Cu in 75% of daily demand from Cu-Gly. OG50—the group received Cu in 50% of daily demand from Cu-Gly. Differences between letters given above columns (**a**, **b**) mean significant differences with *P* < 0.05

The Cu plasma concentration of the rats treated with the organic form of Cu (irrespective of its concentration) was significantly higher than in the control rats administered with S-Cu (Fig. 1A). The Fe (Fig. 1B) and Zn (Fig. 1C) plasma concentrations were similar in the control rats and animals supplemented with the organic form of Cu (regardless of the amount of daily demand) and did not differ between each other (Fig. 1).

### Bone Cu Content

**Fig. 2:**
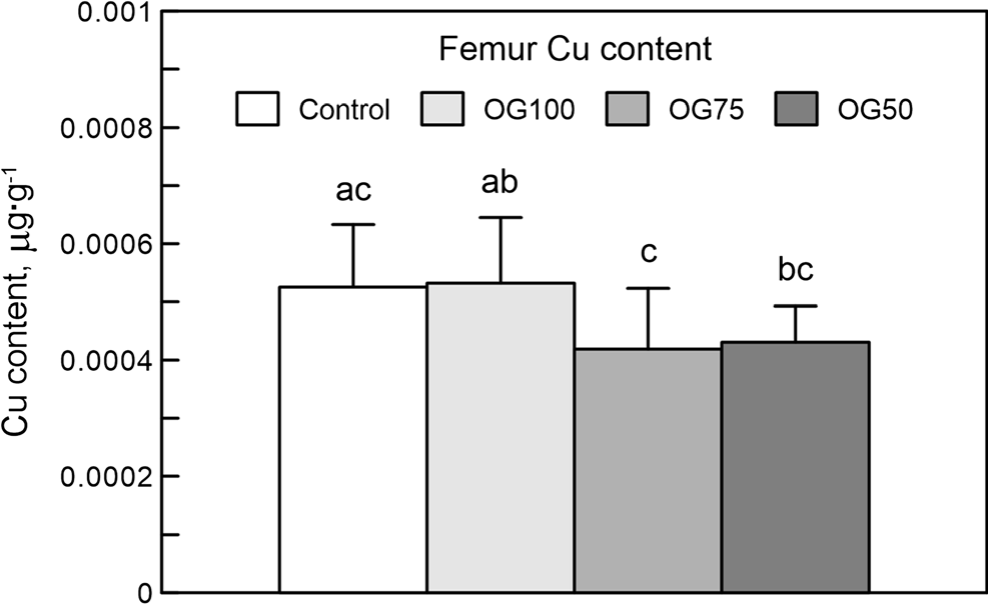
The bone content of copper (Cu) in 24-week-old rats treated with Cu in organic (Cu-Gly) and inorganic form (S-Cu) after 12 weeks of the duration of the study. Data given are mean ± SD, *P < 0.05. Differences between letters given above columns (**a**, **b**, **c**) mean significant differences with P < 0.05. The description of the groups as in the Fig. 1

The bone Cu content in the rats supplemented with the recommended Cu dose, irrespective of its form, was higher than the content exhibited by the other Cu-Gly-treated groups (Fig. 2).

### Bone Morphology, Geometry, and Mechanical Properties

The intake of Cu in the Cu-Gly form, irrespective of its concentration, did not influence the bone mass and length or the mass/length ratio (Table 3). However, Cu-Gly administration in 100% of daily demand decreased all measured diameters; in 75%, it decreased the horizontal internal diameter (h), and in 50%, it decreased both internal diameters (h, b), compared to the control group (S-Cu). Moreover, Cu-Gly influenced the horizontal internal diameter, irrespective of its concentration, while its effect on the other diameters was dependent on the concentration (Table 3). Further, Cu-Gly increased the cortical index, irrespective of its concentration. An increase in the cross-section area was observed in the OG50 group, and higher MRWT was found in the OG100 and OG50 groups. On the other hand, a decrease in the values of the moment of inertia was noted in the OG100 group, and a lower index of gyration was calculated in the OG100 and OG50 groups, compared to the other groups (Table 3).

**Table 3:** Physical, mechanical, and geometric properties of the femur obtained from 24-week-old rats

Item	Group				SEM	*P* value
	CONT (*n* = 12)	OG100 (*n* = 12)	OG75 (*n* = 12)	OG50 (*n* = 12)		
Bone general properties						
Bone mass (g)	1.22 ± 0.32	1.21 ± 0.07	1.27 ± 0.16	1.25 ± 0.01	0.027	0.825
Bone length (mm)	38.20 ± 3.66	39.50 ± 0.53	39.0 ± 1.05	38.45 ± 0.52	0.298	0.438
Mass/length ratio	0.031 ± 0.006	0.032 ± 0.002	0.032 ± 0.003	0.032 ± 0.001	0.001	0.489
Bone geometrical properties						
Horizontal internal diameter (h) (mm)	2.72^a^ ± 0.300	2.15^b^ ± 0.36	2.24^b^ ± 0.06	2.31^b^ ± 0.09	0.049	<0.001
Horizontal external diameter (H) (mm)	4.89^a^ ± 0.32	4.33^b^ ± 0.23	4.61^a,b^ ± 0.32	4.71^a^ ± 0.21	0.052	<0.001
Vertical internal diameter (b) (mm)	1.93^a^ ± 0.25	1.57^b^ ± 0.06	1.88^a^ ± 0.08	1.71^b^ ± 0.07	0.030	<0.001
Vertical external diameter (B) (mm)	3.53^a^ ± 0.25	3.18^b^ ± 0.08	3.40^a^ ± 0.19	3.49^a^ ± 0.112	0.034	<0.001
Cross-section area (A) (mm^2^)	9.38^a^ ± 1.51	8.15^a^ ± 0.37	9.45^a^ ± 1.73	11.56^b^ ± 1.30	0.281	<0.001
Mean relative wall thickness (MRWT)	0.81^a^ ± 0.24	1.03^b,c^ ± 0.12	0.98^a,b^ ± 0.15	1.20^c^ ± 0.10	0.033	<0.001
Cortical index (CI) (%)	43.62^a^ ± 6.96	50.45^b^ ± 3.11	49.30^b^ ± 4.00	53.73^b^ ± 1.46	0.869	<0.001
Midshaft volume	1.44 ± 0.29	1.29 ± 0.04	1.47 ± 0.28	1.51 ± 0.14	0.034	0.128
Moment of inertia (Ix)	9.50^a^ ± 2.31	6.55^b^ ± 0.79	9.20^a^ ± 2.42	9.94^a^ ± 0.99	0.338	<0.001
Index of gyration (Rg)	1.00^a^ ± 0.05	0.89^b^ ± 0.03	0.98^a^ ± 0.04	0.93^b^ ± 0.01	0.008	<0.001
Bone mechanical properties						
Ultimate strength (N)	143.6^a^ ± 25.6	177.0^b^ ± 1.0	202.0^c^ ± 33.7	147.8^a^ ± 13.6	4.749	<0.001
Max. elastic strength (N)	80.0^a^ ± 14.5	125.0^b^ ± 5.3	155.0^c^ ± 15.8	79.1^a^ ± 10.4	5.379	<0.001
Sigma elastic	60.8^a^ ± 19.9	121.9^b^ ± 18.4	118.7^b^ ± 13.3	54.8^a^ ± 13.9	5.555	<0.001
Sigma max	106.9^a^ ± 25.6	172.1^b^ ± 19.8	152.8^b^ ± 8.7	101.9^a^ ± 22.0	5.607	<0.001
Bending moment	3.04^a^ ± 0.61	4.94^b^ ± 0.27	6.06^c^ ± 0.78	3.05^a^ ± 0.44	0.219	<0.001

Data given are mean ± SD. CONT—the control group received Cu in 100% of daily demand from sulfate (S-Cu), OG100—the group received Cu in 100% of daily demand from Cu-Gly, OG75—the group received Cu in 75% of daily demand from Cu-Gly, OG50—the group received Cu in 50% of daily demand from Cu-Gly

^a,b, c^Mean values in rows with different letters differ significantly at *P* < 0.05

The Cu-Gly-treatment in 100 and 75% of daily demand resulted in an increase in all mechanical parameters (Table 3).

### Bone Histomorphometry

Microscopic assessment of cancellous bone in rats supplemented with the organic form of Cu (regardless of the amount of daily demand) showed a significant increase in the real bone volume in the epiphysis, compared to the control supplemented with S-Cu (Table 4). Moreover, the Cu-Gly supplementation in the OG50 group resulted in an increase in the mean trabecular thickness in the epiphysis, compared to the other groups. Additionally, a decrease in the mean and maximal trabecular space in the OG100 and OG50 groups and in the mean maximal trabecular space in the OG75 group was noted, compared to the S-Cu-treated group (Table 4).

**Table 4:** Histomorphometrical parameters of trabeculea of cancellous bone in the femur obtained from 24-week-old rats

Item	Group				SEM	*P* value
	CONT (*n* = 12)	OG100 (*n* = 12)	OG75 (*n* = 12)	OG50 (*n* = 12)		
Femur epiphysis						
BV/TV (%)	32.08^a^ ± 2.04	50.06^b^ ± 9.89	50.04^b^ ± 3.89	53.06^b^ ± 7.02	1.629	<0.001
Tb.Th. mean (μm)	39.81^a^ ± 3.68	39.96^a^ ± 12.75	40.28^a^ ± 3.89	54.97^b^ ± 10.84	1.692	<0.001
Tb.Th. max (μm)	126.2 ± 7.8	116.4 ± 29.5	118.8 ± 44.9	150.2 ± 36.3	5.371	0.085
Tb.Sp. mean (μm)	170.0^a^ ± 14.0	84.1^c^ ± 6.72	102.1^c,b^ ± 19.1	104.2^b^ ± 18.8	5.615	<0.001
Tb.Sp. max (μm)	373.6^a^ ± 1090.1	231.03^b^ ± 31.0	318.9^a,b^ ± 83.6	247.6^b^ ± 68.6	14.788	<0.001
Femur metaphysis						
BV/TV (%)	36.48^b^ ± 2.30	30.11^a^ ± 4.13	39.22^b^ ± 3.63	33.88^a^ ± 1.73	0.766	<0.001
Tb.Th. mean (μm)	63.48^a^ ± 3.81	49.16^b^ ± 10.01	52.18^b^ ± 2.58	51.30^b^ ± 7.69	1.337	<0.001
Tb.Th. max (μm)	158.4^a^ ± 7.5	139.2^a,b^ ± 35.3	115.4^b^ ± 9.8	117.5^b^ ± 20.4	4.227	<0.001
Tb.Sp. mean (μm)	153.1^a^ ± 11.9	276.5^c^ ± 32.6	175.7^a^ ± 13.2	245.1^b^ ± 12.9	8.391	<0.001
Tb.Sp. max (μm)	304.8^a^ ± 61.6	500.3^c^ ± 98.3	308.7^a^ ± 38.8	384.4^b^ ± 17.4	15.403	<0.001

Data given are mean ± SD. CONT—the control group received Cu in 100% of daily demand from sulfate (S-Cu), OG100—the group received Cu in 100% of daily demand from Cu-Gly, OG75—the group received Cu in 75% of daily demand from Cu-Gly, OG50—the group received Cu in 50% of daily demand from Cu-Gly

^a,b,c^Mean values in rows with different letters differ significantly at *P* < 0.05

Further, a decrease in the real bone volume in the metaphysis linked with a decrease in the mean trabecular thickness was observed in the OG100 group, compared to the control group (S-Cu). Additionally, the highest increase in the trabecular space was observed in this group, compared to the other groups (Table 4). Moreover, the Cu-Gly-treatment in 50% of daily demand resulted in a decrease in the bone volume connected with decreased trabecular thickness and increased space in the metaphysis, compared to the control group (Table 4). However, no decrease in the real bone volume was observed in the OG75 group, but a decrease in the thickness of trabeculae was noted in this group, compared to the control group (S-Cu) (Table 4).

### Morphology of Articular and Growth Plate Cartilages

**Fig. 3:**
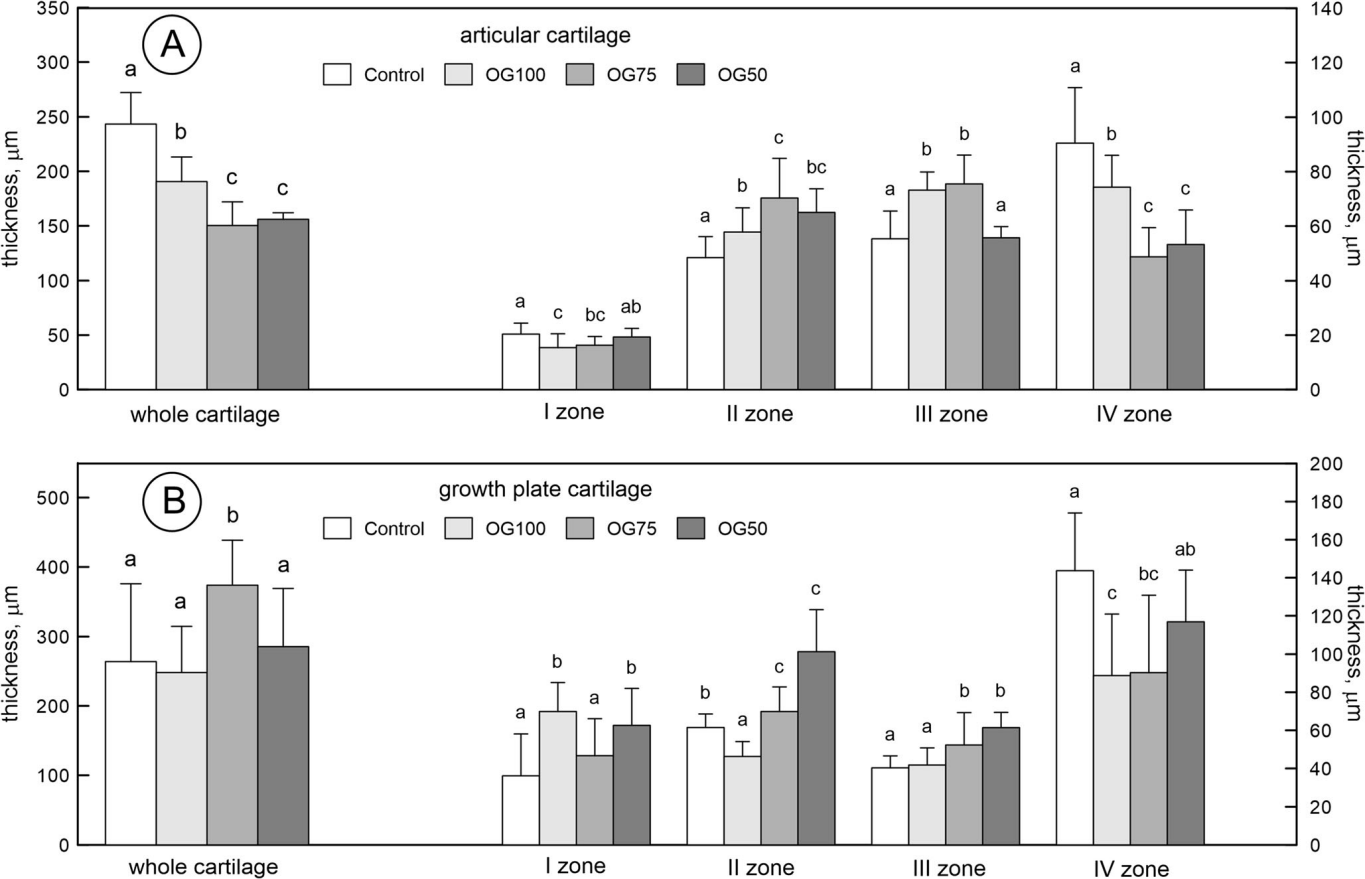
The morphology of articular cartilage (**A**) and growth plate (**B**) of the femur obtained from 24-week-old rats treated with Cu in organic (Cu-Gly) and inorganic form (S-Cu) after 12 weeks of the duration of the study. The description of the groups as in the Fig. 1

The Cu-Gly-treatment (regardless of its concentration) significantly decreased the total thickness of the articular cartilage and increased the thickness of zone II, compared to the control group supplemented with S-Cu (Fig. 3A). On the other hand, the influence of Cu-Gly on the thickness of zone I, III, and IV was concentration dependent. The decrease in zone I and the increase in zone III were noted in the OG100 and OG75 groups, compared to the other groups, while the thickness of zone IV decreased in the OG100 group, compared to the S-Cu-treated group and increased compared to the other Cu-Gly-treated groups (Fig. 3A).

The Cu-Gly-treatment in the OG75 group significantly increased the total thickness of the growth plate compared to other groups (Fig. 3B). The Cu-Gly-treatment in OG100 and OG50 groups significantly increased the thickness of zone I in the growth plate compared to the other groups. Moreover, the Cu-Gly-treatment in OG75 and OG50 groups significantly increased the thickness of zones II and III in the growth plate compared to the other groups. On the other hand, Cu-Gly in the OG100 decreased the thickness of zone II and IV compared to the other groups. The thickness of the zone IV decreased in the group OG75 compared to the group S-Cu treated (Fig. 3B).

### Proteoglycan Content in Articular Cartilages

**Fig. 4:**
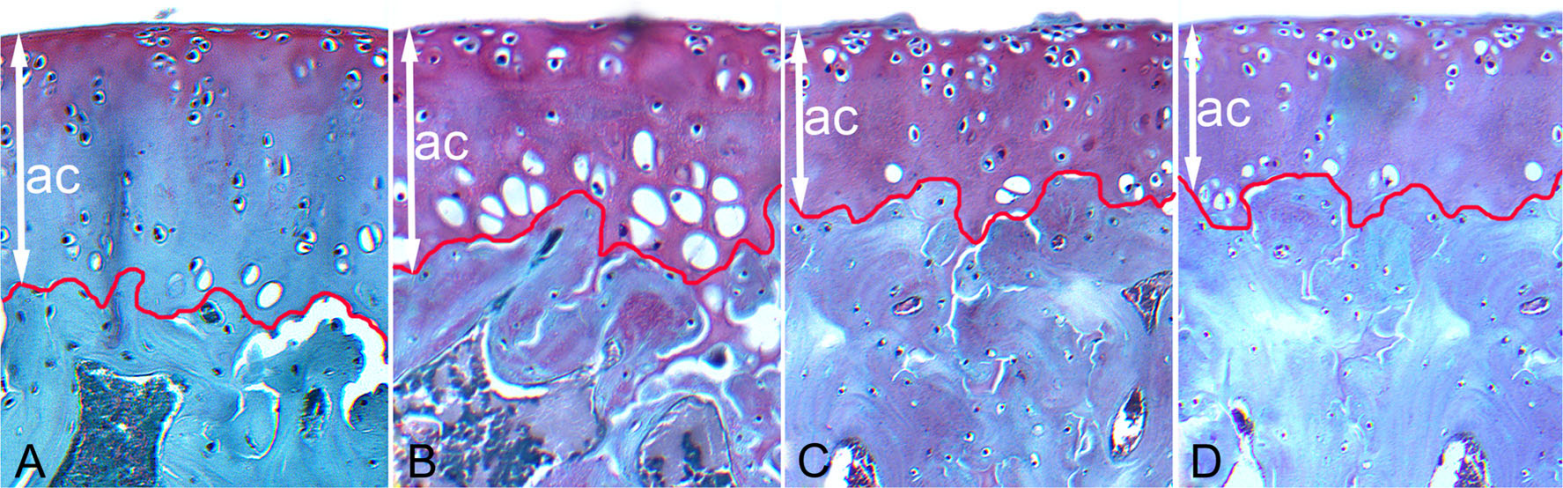
Representative images of safranin-O staining carried out on formaldehyde-fixed sections from the femoral articular cartilage of 24-week-old rats treated with Cu in organic (Cu-Gly) and inorganic form (S-Cu) after 12 weeks of the duration of the study. **a** The control group (S-Cu). **b** 100% Cu-Gly. **c** 75% Cu-Gly. **d** 50% Cu-Gly. The cartilage from the OG50 group displayed a lower proteoglycan content (exhibiting very weak red staining), while the other rats treated with Cu-Gly demonstrated moderate to very strong red staining linked with a high content of proteoglycans. The concentration of proteoglycans exhibited a gradual increase with the distance from the periphery of the cartilage and loss of SO staining in the control rats supplemented with S-Cu. The description of the groups as in the Fig. 1. *Red lines* indicate the bottom border of articular cartilage whereas *white arrow* indicated its thickness. Magnification ×200

Proteoglycan staining with SO showed a lower proteoglycan content (exhibiting very weak red staining) in the cartilage from the OG50 group, while the other rats treated with Cu-Gly demonstrated moderate to very strong red staining linked with a high content of proteoglycans. The concentration of proteoglycans exhibited a gradual increase with the distance from the periphery of the cartilage and loss of SO staining in the control rats supplemented with S-Cu (Fig. 4).

## Discussion

It is known that correct Cu status in living organisms is required for proper growth [19]. A variety of indicators were used to establish the recommended levels for Cu, including plasma Cu concentration, serum ceruloplasmin activity, superoxide dismutase activity, or platelet Cu concentration [7]. However, plasma Cu or ceruloplasmin is a relatively insensitive indicator [20–22]. Our earlier study shows that the Cu liver content (which could be a better indicator of Cu status) is not affected by Cu-deficient diet (Cu-Gly) in adolescent rats [23]. Considering the role of dietary Cu in potentially deficient status, our rats had increased plasma Cu concentrations after the Cu-Gly supplementation, irrespective of the dose used. Additionally, the Fe and Zn concentration was not affected either. This was in agreement with another study, where Zn and Fe absorption and retention were generally not affected by the Cu addition or its sources [24]. Further, in view of the role of dietary Cu in general growth, our rats were characterized by the same water and feed intake. However, the Cu-Gly-treatment increased the body weight of all our rats, irrespective of the Cu-Gly concentration after 6 weeks of administration, but after 12 weeks, the positive effect on final body weight was only noted in the rats supplemented with 75% of daily demand of Cu, compared to the control rats treated with a full dose of S-Cu. A comparison of these findings with those from other studies is somewhat difficult because no studies of the effects of Cu-Gly supplementation at different concentrations below the daily demand are available. In agreement with our data, there is a recent study indicating that an organic form of Cu (Cu citrate) stimulates growth at a lower concentration than that of S-Cu in broiler and swine [22, 25]. Different results were obtained in a study with Cu-deficient chickens, which showed no difference in body weight. Additionally, there was no difference in the basal morphology of the tibia between Cu-deficient and control chickens [26]. There is also one study performed with Cu-Gly added to growing chicken feed mixtures through 6 weeks, covering 100, 50, and 25% of the total requirement of the Cu recommended for broiler chickens, which shows that the reduction of Cu-Gly in feed additive to 25% results in an increase in bone weight but without changes in the length [8].

In our study, there were no changes in bone morphology, but significant alteration was noted in the geometry, which was Cu-Gly-dose dependent. Our rats supplemented with 50% Cu-Gly in the feed additive had less mature bone with a thicker wall. However, it did not influence its mechanical endurance. In the case of the supplementation of 75% Cu-Gly in the diet, it should be considered if the reduction of one internal diameter may have resulted in the increase of the ultimate and maximal elastic strengths by 14 and 24%, respectively, compared to the 100% Cu-Gly supplementation and by 41 and 93%, respectively, compared to the S-Cu group. This was the strongest effect noted among the Cu-Gly-supplemented groups. Although the 100% Cu-Gly administration induced the greatest changes in bone geometry, the alteration in mechanical endurance was manifested less clearly (23% increase in the ultimate force). In general, it may be proved that the supplementation of Cu-Gly even in deficient amounts, compared to the recommended dose, can positively influence the process of bone mineralization or there is an additional mechanism, which should be studied.

Thus, it has been shown that Cu is required not only for proper growth but also for bone strength [19, 27]. Mature bone consists of the bone matrix (an organic phase, which includes collagen type I and noncollagenous proteins) and the mineral phase (hydroxyapatite). The amounts of these various components vary with age, gender, disease, and treatment. It was shown that although the ash weight and the calcium content of the femora from Cu-deficient animals did not differ from those of the controls, mechanical endurance was reduced [28]. There was no effect of Cu, even at an increased intake above the usual dietary intake, on osteocalcin (biomarker of bone metabolism) in healthy young adult females in an over 4-week period [29]. It is likely, therefore, that the impaired mechanical strength was related to defects in the collagen component of the bone [28]. Copper deficiency in man and in animals is associated with bone fragility ascribed to defective cross-links. Collagen cross-links are essential for bone to possess a sufficient deflection capacity, bending strength, and stiffness [30]. As has been mentioned, Cu plays a role in collagen cross-links, i.e., elements of the extracellular matrix. It has been well described that the connective tissue strength is based on cross-links as well as the orientation, density, and length of both collagen fibrils and fibers, and there is a functional link between the skeletal muscle cell and the bone. It is known that the force transmission of the muscle-tendon complex on bone is dependent on the structural integrity between muscle fibers and the extracellular matrix [31]. Other possible mechanisms refer to the role of Cu as an angiogenic factor. Poor vascularization of the cartilage is the characteristic of the lesion [32].

It should be investigated further how our Cu-Gly supplementation in the Cu-deficient diet enhanced mechanical endurance and simultaneously triggered an osteoporotic effect in cancellous bone, especially in the femoral metaphysis. Moreover, it should be answered why the strongest osteoporotic effect in cancellous bone was observed in the group supplemented with Cu-Gly in the recommended daily dose. Although there was no significant effect on final body weight, these rats had longer femora than the other animals; however, this was not statistically proven. Could any role be played by the bone Cu content, which was higher in our rats supplemented with the recommended dose of Cu, compared to those fed Cu-low diet? Moreover, it is interesting in which form copper is retained in the bone. It is a well-known fact that small amounts of certain metal ions can displace calcium from hydroxyapatite. On the other hand, Cu may also be retained by the organic part of the bone. This should be further investigated. However, it has been proven that Cu content in the bone is negatively correlated with bone Ca, bone density, and collagen content in mice, and the bone Cu level in human subjects with osteoporosis is the same or slightly higher than in healthy individuals of the same age [33].

Our study also presented the results of the measurement of the growth plate, which indicated that the Cu-Gly complex (depending on the dose) influenced the growth plate in a different manner. The complex Cu-Gly given in 100% of demand reduced the proliferative zone, which may indicate disturbances in the cell cycle, division, and differentiation accompanied by a decrease in the rate of chondrocyte proliferation, as well as the thickness of bone trabeculae, which became thinner with the highest separation. Cu supplementation in the dose of 75% of demand increased the total thickness and hypertrophy zone; this may suggest a reduced degree of mineralization (reduced zone IV), which depends on the size and number of cells in the hypertrophic zone of the growth plate and the amount of calcium released from the mitochondrion. The dose of 50% of demand caused disturbances in bone growth and influenced its geometry, not only the architecture of the trabeculae. The present study also showed a significant decrease in the articular cartilage thickness in the rats supplemented with Cu-Gly, irrespective of its dose. The shortening of the superficial zone in our rats can change the distribution of the load through the joint with functional consequences. It might trigger the degradation of the articular cartilage, thereby causing difficulties in movement, because the reduction of this zone can change the elasticity of articular cartilage, which contributes to reversibility of the deformation caused by the impact of the load during movement. Thus, the organic form may lead to alterations within the cartilage during normal function and, with time, osteoarthritis can develop. On the other hand, the supplementation of Cu-Gly seemed to enhance the proteoglycan content in articular cartilage (Fig. 4). The degradation of proteoglycans can play a pivotal role in destabilization of the collagen network. Proteoglycans ensure stability of articular cartilage and their content in normal cartilage is directly proportional to the intensity of safranin-O staining, which has been used to demonstrate any changes that occur in articular diseases [34].

However, the level of the chemical Cu form applied in these observations is important, because the reference group was given Cu at the same dose but in the S-Cu form. Copper, when fed at prophylactic concentrations, may function by increasing absorption or by providing growth-promoting effects, possibly by shifting the microbial populations within the gastrointestinal tract [35] or through other unknown mechanisms. Because different Cu sources have different relative bioavailabilities [35], the mechanism and extent of growth-promoting effects are rather varied. Attempts to quantify the bioavailability when fed at marginally deficient concentrations of alternate sources of Cu are numerous. For those particular studies, copper sulfate (S-Cu) has been used as a reference point for comparing the relative bioavailability of various Cu sources [35].

To the best of our knowledge, this is the first study that has examined both the mechanical properties of the bone and histomorphometry of the trabecular bone and hyaline cartilage not only in Cu-deficient diet but also in diet with a similar level of Cu given in a different source.

## Conclusions

No studies conducted so far have provided a detailed morphological analysis of the growth plate, cancellous bone, and articular cartilage of adult rats administered with diet containing different Cu forms. Moreover, the Cu-Gly treatment influenced the trabecular architecture and morphology of the growth plate in dose-dependent manner. However, dietary Cu given to adult rats in the Cu-Gly complex covering the daily demand in 75% exerted a positive effect on bone metabolism and appeared to be the most effective among the investigated doses of the organic form.
